# Colour information biases facial age estimation and reduces inter-observer variability

**DOI:** 10.1038/s41598-023-39902-z

**Published:** 2023-08-14

**Authors:** Jean Y. J. Hsieh, W. Paul Boyce, Erin Goddard, Colin W. G. Clifford

**Affiliations:** School of Psychology, UNSW Sydney, Sydney, NSW 2052 Australia

**Keywords:** Psychology, Human behaviour

## Abstract

Age estimation is a perceptual task that people perform automatically and effortlessly on a daily basis. Colour has been identified as one of the facial cues that contributes to age perception. To investigate further the role of colour in age perception, we manipulated the chromatic content of facial images holistically. In Experiment 1, images were shown in colour or grey scale; in Experiment 2, images were shown with red–green contrast increased or decreased; in Experiment 3, images were shown with modified yellow–blue contrast. We examined whether the presence of chromatic information biases the perception of age and/or affects inter-observer variability in age judgements, and whether specific chromatic information affects the perception of age. We found that the same face tended to be judged as younger with increased red–green contrast compared to decreased red–green contrast, suggesting that red–green contrast directly affects age perception. Inter-observer variability in age ratings was significantly lower when participants were asked to rate colour compared with grey scale versions of images. This finding indicates that colour carries information useful cues for age estimation.

## Introduction

Age is an important social attribute, making age estimation an ecologically relevant component of face perception. It determines rights, responsibilities and even social status^1^. However, perceived age does not always reflect an individual's biological age, since facial appearance is also influenced by environmental and lifestyle factors^1^. A longitudinal study of Danish twins reported that perceived age is a better biomarker of health than chronological age due to the influence of lifestyle and social factors^2^. This social relevance is also reflected in most people wanting to be perceived as younger, since looking youthful is associated with health and attractiveness^3–5^. Additionally, researchers have also found that attractive people are judged and treated more positively than unattractive people^6^.

The eyes and lips have been reported to be particularly relevant to estimating age compared to other facial features. The surrounding area, shape, and size of the eyes and sclera colour have been shown to help people make a judgement of age^7^. Lips are also potentially a reliable cue to age since their volume^8^ and redness^9^ decrease significantly with age. Gunn et al.^8^ conducted a study to investigate the factors that contribute to a youthful appearance. They collected ratings from participants on perceived age, attractiveness, and health based on images of females ranging in age from 16 to 96 years old. The study found that lip height, skin wrinkling, and hair greying were all significant factors that were independently associated with how old the women appeared for their age.

Besides facial features, there are other surface-based cues associated with perceived age^10–12^. These cues can be divided into two categories. The first category, known as “pigmentation,” includes variations in skin tone and texture^10^. Faces with uneven skin tone are more likely to be judged as older compared to those with even skin tone^8^. Sun exposure is also associated with a higher perceived age^13,14^ as it causes uneven skin tone^15^ and wrinkling^8^. The second category is the pattern of shading and shadow created by the three-dimensional (3D) shape of the face^10,11,16^. For example, nose shape changes significantly with age^17^, but it is not yet clear if people use the nose as a cue to estimate age. Empirically, digitally reducing skin wrinkles, skin sagging, or skin unevenness has been shown to give a more youthful appearance^15,18^.

Colour information of the face has not only been reported to be important in the perception of age^15,19^, but also to contribute significantly to perceptions of sex/gender and ethnicity^16,20^, health^7,15,21^, attractiveness^21,22^, and identity^23^. When investigating age perception, Fink et al.^18^ applied skin colour distributions from different ages to identical 3D shape-standardised female faces. Participants rated age, health, and attractiveness of the 3D rendered faces. Faces with younger surface-based information were perceived as younger and received significantly higher ratings for attractiveness and health. Analysis of the images revealed that the skin colour distribution of younger faces was more even compared to older faces. Fink et al.^18^ suggested that skin colour distributions provide information independent of facial form and skin surface topography that contributes to the perception of female facial age and judgements of attractiveness and health. However, further research is needed to determine if these findings can be applied to actual face images. Therefore, we intend to explore this topic further in our proposed study.

Facial contrast, defined as the contrast between facial features such as the eyes and lips and the surrounding skin, is a potential cue for age estimation^24,25^. It should be noted that this is different from ‘skin contrast’ as defined by other researchers^15^. Studies have found that female faces have greater facial contrast than male faces, and facial contrast plays an important role in sex/gender classification and attractiveness^22,25^. Porcheron et al.^24^ proposed that facial contrast decreases with age and serves as a cue for identifying age. They measured the facial contrast of Caucasian female faces within the CIE L*a*b* colour space (Lab space) and asked participants to estimate age from the original and contrast-manipulated images. Female face images were judged to be significantly younger when shown with greater a* (red–green) contrast around the mouth, greater luminance contrast around the eyes, or greater luminance contrast around the eyebrows. It is worth noting that these estimates of facial contrast did not distinguish between skin pigmentation and luminance cues related to topographic factors, and that measuring facial contrast required individual labelling of facial regions of interest, which could make standardisation difficult. Overall, these findings suggested that colour contrast both within features/skin and between features is a potential cue to age.

A key challenge for facial age estimation research is the fact that faces are complex stimuli, and the space of possible variables driving age perception is large. A common approach is to manipulate specific facial features and observe changes in age ratings to identify relevant features^18,24^. While this approach is valid, it relies on the researcher first identifying candidate facial features, leaving open the possibility that more complex or subtle features are missed. To circumvent this limitation, we proposed to manipulate the colour contrast of entire face images using the three dimensions of Lab space, as opposed to manipulating specific facial features. We hypothesised that faces would be perceived as younger while increasing both the level of redness^18,24^ and yellowness. Yellowness is associated with skin health^26,27^ and health is a highly associated with age^2^. Whether colour rather than grey scale presentation influences the perception of age is unknown.

## Results

### Experiment 1

We tested whether colour rather than grey scale presentation systematically biased the perception of age, or affected inter-observer variability in age judgements. We conducted data cleaning before running any analyses. There were 7800 ratings collected from 39 included participants. From these, we excluded ratings under 13 years since images from the Chicago face dataset (CFD) are all images of adults. We also excluded absolute deviations from the CFD average rated age larger than 25. By doing so, 32 of 7800 trial ratings were excluded.

**Figure 1:**
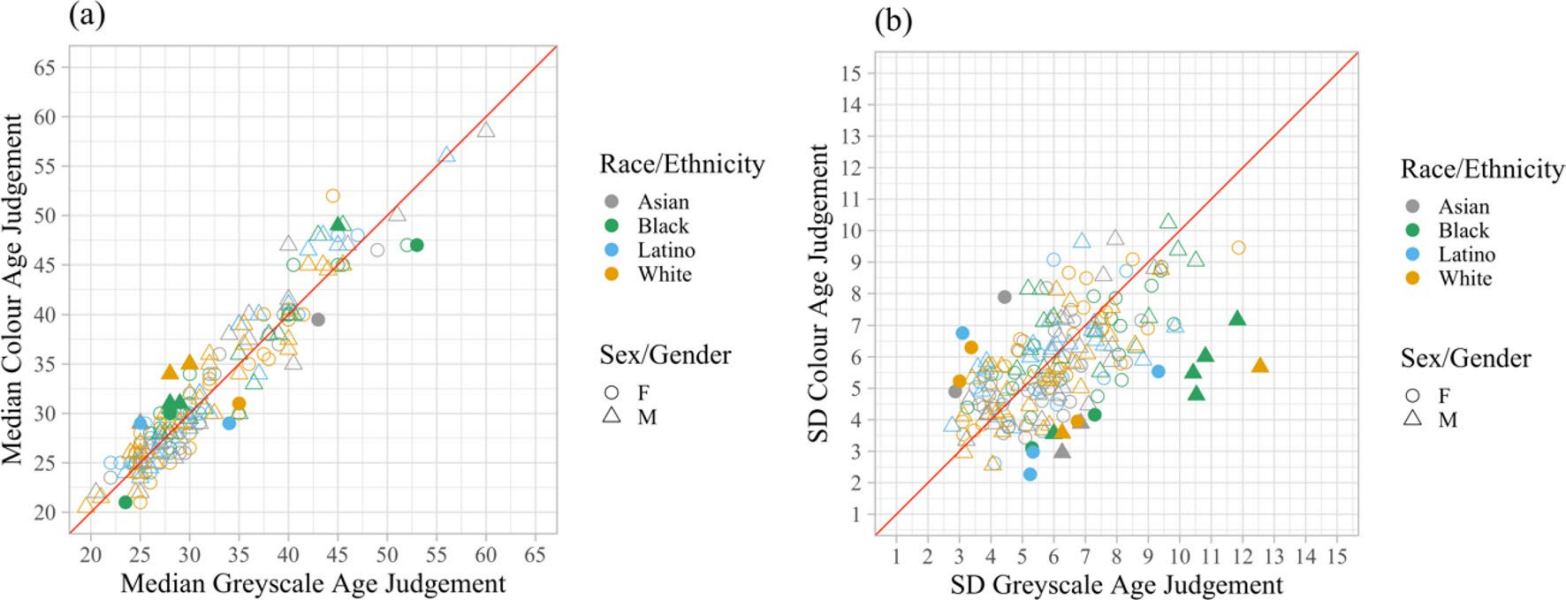
Rating comparison in Experiment 1. *Note*: Each of the 200 data points represents a particular stimulus identity. The red line is a reference line of equality. The legends describe the characteristics of the images. (**a**) Median age ratings. Points above the red line are images that have older ratings in the colour condition compared to the grey scale condition. Filled points are images that have significantly different mean age ratings between the two conditions (independent samples *t* tests, uncorrected for multiple comparisons). No cases exceed the false discovery rate (FDR) threshold for q < 0.05 when we corrected these *p* values for multiple comparisons across images ^28^. (**b**) Between participants *SD* of age ratings. Points above the red line are images that have higher *SD* in the colour condition compared to the grey scale condition. Filled points are images that have significantly different *SD*s between the two conditions (*F*-tests, uncorrected for multiple comparisons). 4 cases exceed the false discovery rate (FDR) threshold for q < 0.05.

To establish if presentation in colour versus grey scale systematically biases ratings of age, we compared the median rating of each image in colour (*M* = 32.54, *SD* = 9.47) and grey scale (*M* = 32.37, *SD* = 9.64). A paired-sample *t* test indicated there was no significant difference between image conditions (*t*_199_ = 1.37, *p* = 0.19; Fig. 1a).

The standard deviation (*SD*) in ratings gives a measure of inter-observer variability, which reflects the degree of agreement among independent observers, for each image in each of the two conditions. Specifically, the lower the *SD*, the lower the variability and the higher the agreement. To investigate whether image condition affected inter-observer variability, we compared *SD*s of the rating of each image in the colour (*M* = 5.74, *SE* = 1.61) and grey scale (*M* = 6.06, *SE* = 1.96) conditions. A paired-sample *t* test revealed that inter-observer variability was significantly higher in the grey scale condition than in the colour condition (*t*_199_ = − 2.65, *p* = 0.009, Fig. 1b). Inspection of the data indicates an ethnicity effect, such that there is a disproportionately high number of black faces among those that have a significantly higher SD in grey scale conditions (Fig. 1b). A post-hoc chi-squared test confirmed that the ethnicities of these images did indeed differ from the expected distribution, *X*^2^ (3, *N* = 200) = 8.63, *p* = 0.05.

### Experiment 2

**Figure 2:**
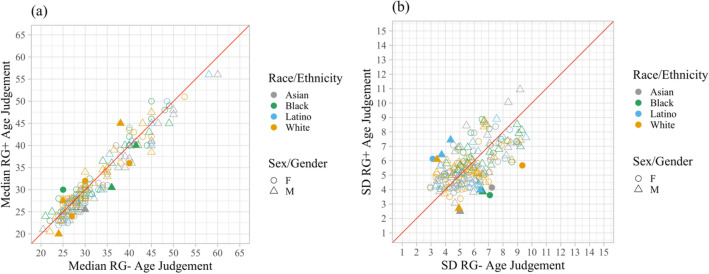
Rating comparison in Experiment 2. *Note*: Each of the 200 data points represents a particular stimulus identity. The red line is a reference line of equality. The legends describe the characteristics of the images. (**a**) Median age ratings. Points above the red line are images that have older ratings in the increased red–green contrast (RG+) condition compared to the decreased red–green contrast (RG−) condition. Filled points are images that have significantly different mean age ratings between the two conditions (independent samples *t* tests, uncorrected for multiple comparisons). No cases exceed the false discovery rate (FDR) threshold for q < 0.05 when we corrected these *p* values for multiple comparisons across images. (**b**) Between participants *SD* of age ratings. Points above the red line are images that have higher *SD* in the RG + condition compared to the RG- condition. Filled points are images that have significantly different *SD*s between the two conditions (*F*-tests, uncorrected for multiple comparisons). No cases exceed the false discovery rate (FDR) threshold for q < 0.05 when we corrected these *p* values for multiple comparisons across images.

We further investigated if facial age estimation is affected by specific chromatic information, specifically red–green contrast. There was a total of 8200 ratings collected from 41 included participants. Using the same data cleaning criteria as in Experiment 1, we excluded 20 of 8200 ratings. To clarify if presentation in increased versus decreased red–green contrast systematically biases ratings of age, we compared the median rating of each image in the increased (*M* = 31.99, *SD* = 9.31) and decreased red–green contrast conditions (*M* = 32.57, *SD* = 9.41) with a paired-sample *t* test (*t*_199_ = 4.46, *p* < 0.001). There was a significant difference between image conditions such that faces tend to be rated younger when presented in the higher red–green contrast condition (Fig. 2a).

We also investigated whether image conditions affected inter-observer variability. A comparison of the *SD* of the rating of each image in both lower red–green contrast (*M* = 5.78, *SE* = 1.54) and higher red–green contrast (*M* = 5.64, *SE* = 1.42) condition with a paired-sample *t* test (*t*_199_ = 1.36, *p* = 0.17) revealed no significant difference for inter-observer variability (Fig. 2b*)*.

### Experiment 3

**Figure 3:**
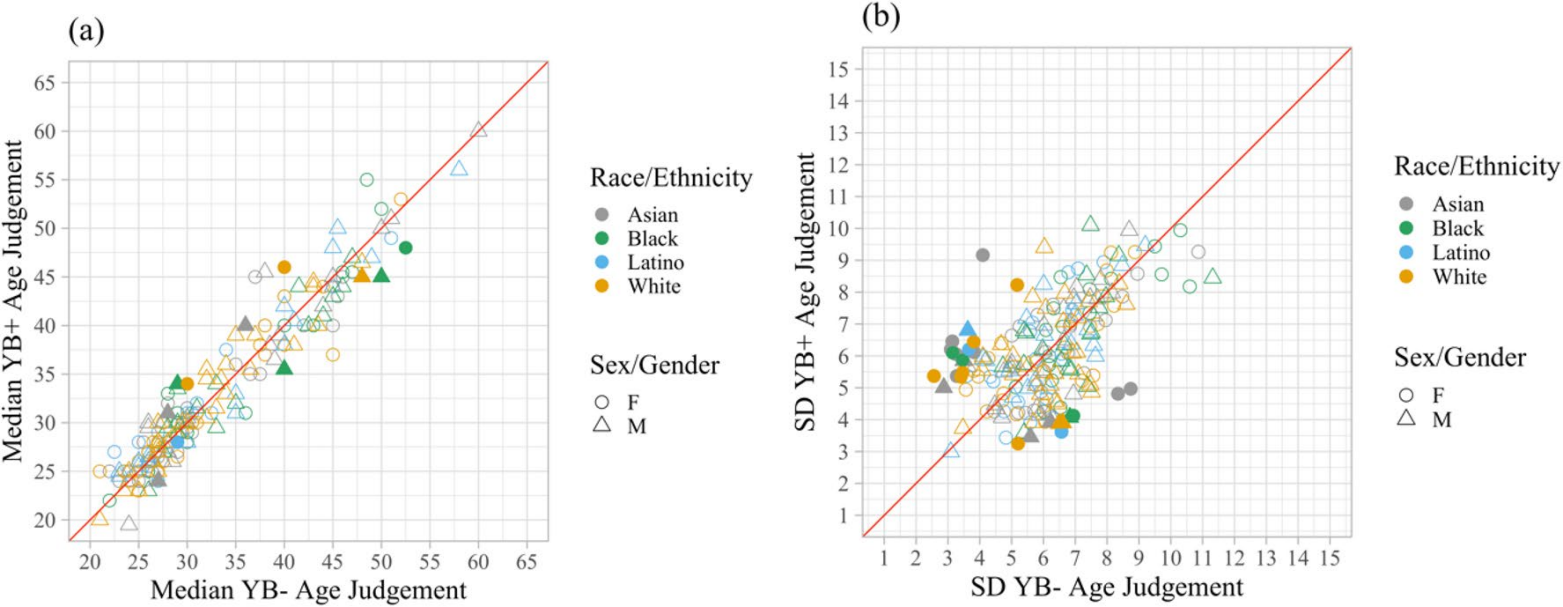
Rating comparison in Experiment 3. *Note*: Each of the 200 data points represents a particular stimulus identity. The red line is a reference line of equality. The legends describe the characteristics of the images. (**a**) Median age ratings. Points above the red line are images that have older ratings in the higher yellow–blue contrast (YB+) condition compared to the lower yellow–blue contrast (YB−) condition. Filled points are images that have significantly different mean age ratings between the two conditions (independent samples *t* tests, uncorrected for multiple comparisons). No cases exceed the false discovery rate (FDR) threshold for q < 0.05 when we corrected these *p* values for multiple comparisons across images. (**b**) Between participants *SD* of age ratings. Points above the red line are images that have higher *SD* in the YB+ condition compared to the YB− condition. Filled points are images that have significantly different *SD*s between the two conditions (*F*-tests, uncorrected for multiple comparisons). No cases exceed the false discovery rate (FDR) threshold for q < 0.05 when we corrected these *p* values for multiple comparisons across images.

We further investigated if facial age estimation is affected by yellow–blue contrast. There was a total of 8600 ratings collected from 43 included participants. Using the same data cleaning criteria as in Experiment 1, we excluded 57 of 8600 ratings. Comparing the increased (*M* = 33.18, *SD* = 9.91) and decreased yellow–blue contrast conditions (*M* = 33.28, *SD* = 10.13), the paired-sample *t* test on the median rated age (*t*_199_ = − 0.50, *p* = 0.62) was not significant (Fig. 3a).

We also investigated whether image conditions affected inter-observer variability. A comparison of the *SD* of the rating of each image in the lower yellow–blue contrast (*M* = 6.15, *SE* = 1.62) and higher yellow–blue contrast (*M* = 6.18, *SE* = 1.58) conditions with a paired-sample *t* test (*t*_199_ = − 0.27, *p* = 0.79) revealed no significant difference for inter-observer variability (Fig. 3b).

## Discussion

The aim of the current study was to establish whether colour and chromatic contrast contribute to facial age judgements. We examined whether colour information biases the perception of age, or affects inter-observer variability in age judgements. Additionally, we also examined which specific chromatic channel affects the perception of age. In Experiment 1, participants were asked to rate images in either colour or grey scale version. The inter-observer *SD* of ratings for individual images was found to be significantly lower for colour presentation. This implies that the participants' responses were more consistent when the images were presented in colour. Comparing median ratings within Experiment 2, we found that face images with higher red–green contrast were rated significantly younger. This finding demonstrates that red–green contrast can directly influence the perception of age such that the same face tends to be rated as younger with increased compared to decreased red–green contrast. No significant effect was found while manipulating yellow–blue contrast in Experiment 3.

**Figure 4:**
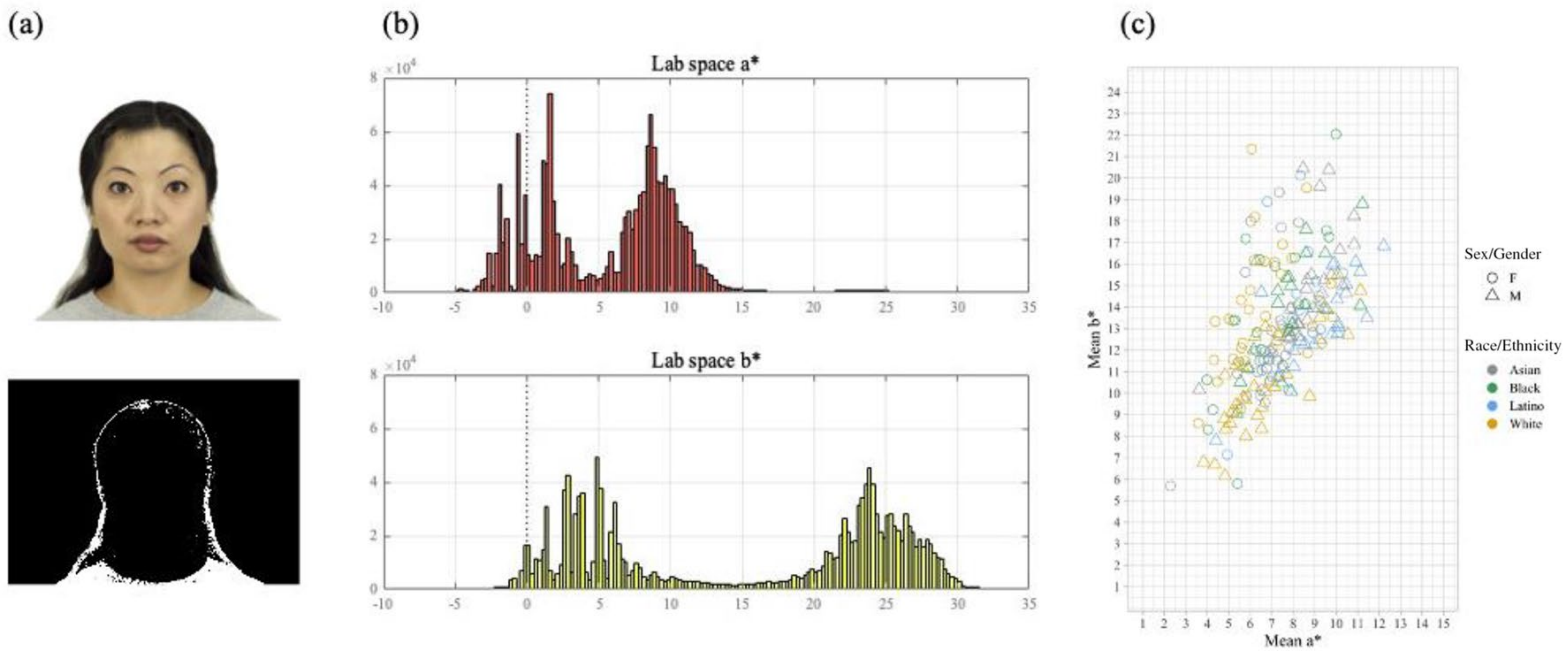
Face colour in Lab space. *Note*: The example image was drawn from the Chicago Face Database ^29^. (**a**) Example stimulus image and map of pixels where a* < 0. (**b**) Pixel counts of Lab space a* and b* values for this image. Most pixels are red (a* > 0) and yellow (b* > 0). (**c**) Mean chromaticity of face images in Lab space. Each of the 200 data points represents a particular stimulus image. All have mean a* and mean b* greater than zero.

Recent studies have suggested that redness is associated with the perception of various facial attributes, such as age, attractiveness, and health. However, it is unclear whether hues^22^ or colour contrasts^24^ are driving the results. Representing face images in Lab space, it is evident that there is a predominance of red (> 0) and yellow (> 0); there is very little green and even less blue (Fig. 4). Across 200 images, 81.64% of pixels had a* > 0 and 91.52% of pixels had b* > 0. Thus, by manipulating different Lab space contrasts, we were mainly manipulating “redness” and “yellowness” in the facial images. Specifically, increasing red–green contrast increased redness while increasing yellow–blue contrast increased yellowness.

In Experiment 1, we compared the median and *SD* of ratings between colour and grey scale images. We found that the inter-observer *SD* of ratings for individual images was significantly lower for colour presentation. That is, when images were in colour, the participants’ responses were less varied, compared to when the images were presented in grey scale. While we found no evidence that the simple presence or absence of colour systematically affects our perception of age, this finding indicates that chromatic information provides extra information about age, beyond that carried by the achromatic content of the image, and that this is used when making age judgements, in a way that is broadly consistent across observers.

A chi-squared test revealed an imbalance in the ethnicity of faces that have significantly different *SD*s between the two conditions (Fig. 1b), primarily due to a higher number of black faces. All black faces where the difference was significant had a higher *SD* in the grey scale condition, suggesting that colour may influence the perceived age of black faces more than other ethnicities. Additionally, these findings align somewhat with the previously reported ‘other-race effect’, where age estimation is more accurate for individuals of the same ethnicity^30^ and higher familiarity^30,31^. A study in Belgium revealed that Caucasian participants performed better in evaluating ages for Caucasian faces compared to African faces, while African participants performed equally well with both face types, possibly due to the African participants’ time of residence in Belgium^30^. Although our experiment did not control participant ethnicity, the observed differences may be attributable to the unfamiliarity of our primarily domestic student participants with Afro-American black faces, which are underrepresented in Sydney's otherwise diverse racial profile. However, it is notable that in our study we did not observe a significant other-race effect per se, but rather a race-specific decrement in performance when colour was removed from the images, suggesting that processing the age of other-race faces may be disproportionately affected when image information is impoverished. Further investigation should explore the impact of observers’ ethnicity and time of residence in Australia, as well as other means of image degradation, for a more comprehensive understanding.

In Experiment 2, we found that increasing red–green contrast (effectively increasing redness) made faces look younger*,* which complements previous research. Previous studies have shown that increasing the redness of particular facial features (e.g., lips, skin) makes faces look younger^14,15,18,24^. While youthfulness is often associated with health and attractiveness, increasing redness can also make faces look healthier^32,33^ or more attractive^15,18,22^. Facial redness is an indicator of the level of blood oxygenation, which may be interpreted by observers as a cue to underlying physiological health or quality^21,32^.

In Experiment 3, we did not find any evidence that yellow–blue contrast (effectively increasing yellowness) is associated with facial age perception. Furthermore, the variance of ratings between the two conditions did not exhibit any significant differences. Despite evidence that increased yellowness is related to health, which is itself strongly associated with age, we found no evidence that yellowness has any impact on the perception of age in faces. Previous studies have suggested that the b* component of skin colour is greatly influenced by two pigments—melanin and primarily carotenoids^27^. Although carotenoids can serve as a cue for perceiving health, they may not be a reliable indicator of age perception, which could explain the non-significant results obtained in this experiment.

The current study has several limitations that should be considered. Firstly, the CFD images did not come with actual ages, and all selected images used were limited to between 20 and 40 years old based on the subjective age ratings provided, meaning that the findings may not generalise to other age groups^34^. Additionally, the chromatic information used in the images did not isolate surface-based cues, as shading caused by facial topography may have influenced chromatic content. Even though the facial bones almost stop growing after the age of 20, increasing age leads to a dissipation of the soft tissue in the face resulting in the bony appearance of the face in older adults^35^. Nose shape^17^ and the thickness of the soft-tissue^36^ change significantly with age. Such changes in facial topography may cause differential shading that influences the chromatic content of facial images. Therefore, while the current study sheds light on the role of colour and chromatic contrast in facial age perception, it is important to recognise the limitations associated with the age range of face stimuli and the complexity of chromatic information in facial images.

Furthermore, we have an imbalance in the gender distribution of our participants, with a disproportionately low number of male participants. This limits our ability to compare ratings between genders. A previous study has found that when providing age ratings to female faces, female raters tend to be more accurate than male raters^37^. Some researchers have further suggested that this gender effect might be limited to older female faces^38^. However, when both male and female participants were asked to rate faces of both genders^39^, found no significant difference between genders. Given this mixed evidence, exploring gender differences in facial age estimation could be a potential direction for future research.

In summary, our experiments demonstrate that the mere presence of chromatic information reduces inter-observer variability in age judgements and that manipulating red–green contrast biases the perception of age such that redness tends to be associated with youthfulness. Possible directions for future work are to test whether the red–green contrast effect is driven by a hue shift rather than by red–green contrast, and whether the red–green contrast effect generalises to stimuli of a larger age range.

## Methods

### Participants

A different set of participants was recruited for each of the 3 experiments (Table 1 for demographics). All participants had normal or corrected to normal vision. A summary of participant demographics and the stimulus characteristics across all 3 experiments as shown in Table 1. All participants provided informed consent and ethical approval for this study was granted by the UNSW Human Research ethics committee. All methods were performed in accordance with the Declaration of Helsinki. Participants were all UNSW psychology undergraduate students and were recruited through the UNSW SONA system (Research management system) and received course credit.

**Table 1:** Summary of participant demographics and stimulus characteristics.

Experiment	n	Excluded	Included (M/F)	Image conditions
1	40	1	9/30, with a mean age of 19.05 (*SD* = 1.52)	Colour (original)/grey scale
2	45	4	12/29, with a mean age of 20.59 (*SD* = 5.88)	Higher/lower red–green contrast
3	44	1	3/40, with a mean age of 19.05 (*SD* = 1.52)	Higher/lower yellow–blue contrast

### Chicago face database (CFD)

Stimuli were based on 200 full face images from the main CFD set of models recruited in the United States^29^. All selected images showed models photographed with neutral facial expressions, in standardised lighting against a white background. There is no ground-truth age information in the main CFD set, but there are subjective age rating norms for each image (based on n = 1087 ratings). All selected images used were limited between 20 and 40 years old based on subjective age rating norms. We used these average age ratings to identify outliers in our data. Subjective age rating norms for the images were not used for the analysis proper. By self-reported ethnicity, there are images of 60 Asian, 60 White, 40 Black, and 40 Latino faces. The number of self-identified female and male images was balanced for each ethnicity.

### Stimuli

**Figure 5:**
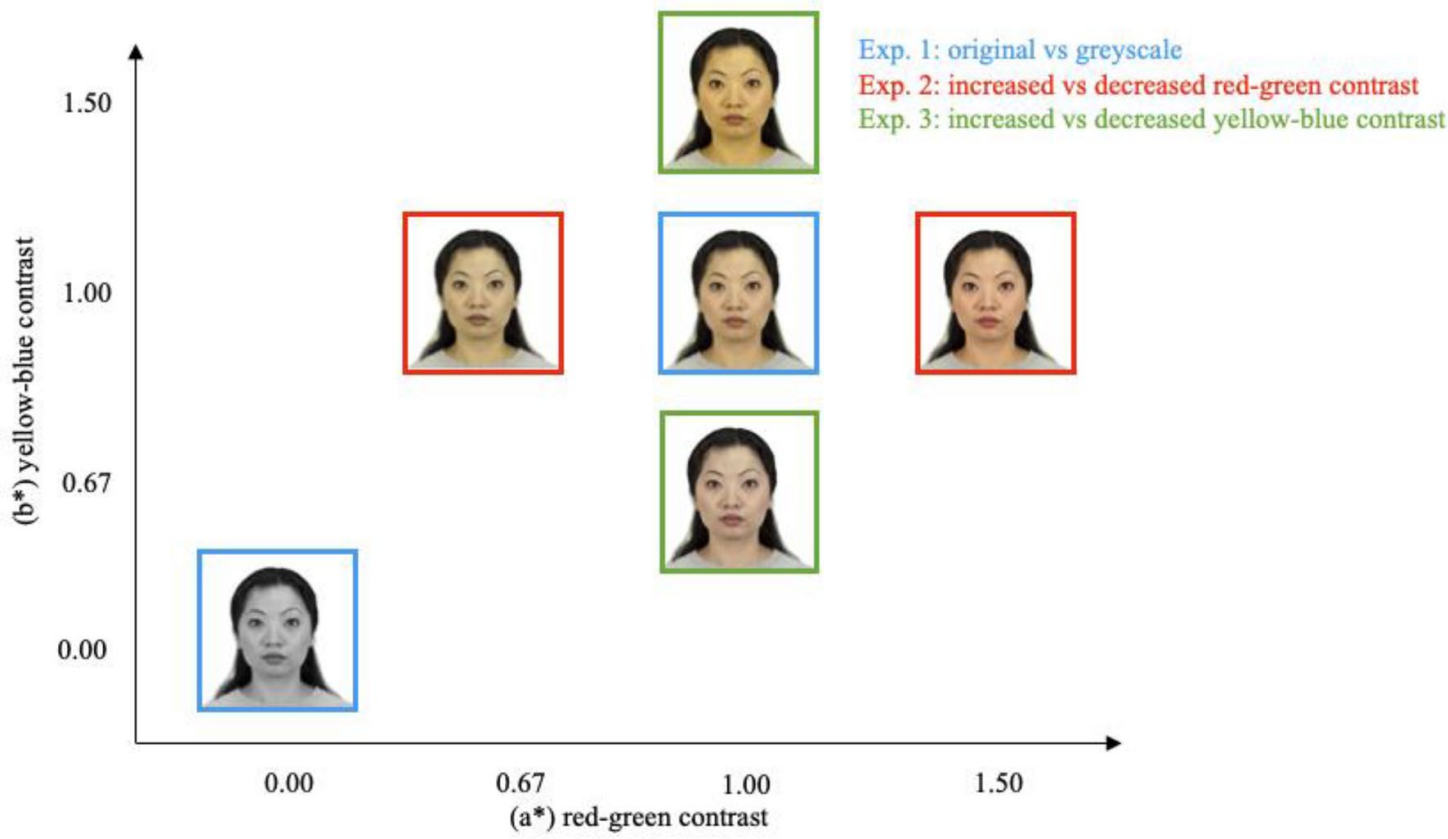
Different manipulations across experiments. *Note*: A sample image from the CFD showing colour manipulations used in Experiments 1–3. The original image is presented in the centre of the graph with a blue frame.

We utilised the CIE Lab* colour space to adjust the colour contrast of the 200 images sourced from CFD. This particular colour space is recognised for its uniform perceptual discriminability, where distances between coordinates of stimuli are predictive of perceived colour difference between the stimuli^40^. The 3 orthogonal dimensions of this colour space are light–dark (L*), red–green (a*), and yellow–blue (b*). Here we manipulated contrast along the chromatic dimensions while keeping the achromatic content (L*) constant. Demonstration images for each experiment are shown in Fig. 5. In Experiment 1, images were shown in original colour or grey scale version; in Experiment 2, images were shown with red–green contrast increased or decreased by a factor of 1.5 while images were shown with similarly modified yellow–blue contrast in Experiment 3. For each image, we first transformed the image from sRGB to CIE Lab, performed the contrast manipulation, then transformed the image back from CIE Lab to sRGB space, using the white point of CIE standard illuminant D65. All image manipulations were conducted using Matlab. The magnitude of the manipulations in Experiments 2 and 3 was based on informal piloting. The difference is perceptible, but the faces still look natural.

### Analyses

In each experiment, a minority of participants gave unreliable ratings and their data were excluded. We excluded participants where the correlation between their ratings and the mean rated ages from CFD was < 0.5. Additionally, we excluded data from individual ratings < 13, as all face images used were of adult faces. Finally, we excluded data where the difference between the referred age from CFD and the rating from participants was > 25. To investigate whether the presentation of images in two different conditions affects the perception of age, we used paired-sample *t* tests to compare the median ratings of each image. We conducted a paired-sample *t* test to examine whether the image conditions had an impact on inter-observer variability by comparing the *SD* of ratings for each image. Furthermore, we performed independent samples *t* tests on each image's median and *SD* to determine if there were any significant effects of presentation condition within the images.

### Procedure and design

This was an online study that allowed participants to join from a personal computer running a Google Chrome browser. We used JATOS^41^ and jsPsych^42^ hosted by a server within the UNSW School of Psychology. Participants were required to run the experiment in full-screen mode. Image size was calibrated to 18 cm (w) × 25.6 cm (h) by adjusting an on-screen rectangle to the size of a credit card prior to the experiment. Participants were asked to rate the age of faces by entering a two-digit integer (between 10 and 99) on the number pad of a computer keyboard. Participants were not informed about the age range of the stimuli and no feedback was given as to the accuracy of their responses. On each trial, an image was presented for 2 s and then removed. A written prompt and a text box would pop up on the screen until the participant made their response. Each participant provided 200 ratings with 100 images presented in colour and 100 in grey scale. Each participant rated each face only once, in colour or grey scale. Stimulus presentation was counterbalanced such that, across participants, each stimulus identity was presented the same number of times in colour and grey scale. Therefore, in Experiment 1, a total of 8000 ratings were collected across all 40 participants. For each stimulus identity, there were 20 ratings in colour and 20 ratings in grey scale, as each participant provided a rating either in colour or in grey scale.

## Data Availability

The datasets generated during the current study are available from the corresponding author on reasonable request.
